# The Relationship between Customers and Community Pharmacies during the COVID-19 (SARS-CoV-2) Pandemic: A Survey from Italy

**DOI:** 10.3390/ijerph18189582

**Published:** 2021-09-11

**Authors:** Francesca Baratta, Michele Ciccolella, Paola Brusa

**Affiliations:** 1 Department of Drug Science and Technology, University of Turin, Via Pietro Giuria 9, 10125 Turin, Italy; paola.brusa@unito.it; 2 FarmaHiSkill Italia, Via Guelfa 5, 40138 Bologna, Italy; m.ciccolella@hiskill.it

**Keywords:** community pharmacies, pharmacists, COVID-19, SARS-CoV-2, Italy, infodemic, syndemic

## Abstract

Community pharmacies are among the most easily accessible health services. Considering the major impact of COVID-19 in social terms, the purpose was to analyse the evolution of the relationship between community pharmacies and customers during the pandemic in 2020 and to understand which strategies should be implemented in the future. The data have been collected from May to December 2020. Pharmacists administered a questionnaire, also available online, to all customers that agreed to participate. The total number of respondents was 502. The results obtained confirm a generally high level of satisfaction with pharmacies among customers and appreciation for the role of community pharmacies. For the future, the priority is to monitor the situation to break down social inequalities. A task that can be entrusted to the branch of the healthcare service ideally suited to this end: local medicine, of which the community pharmacy is an essential element. The post-pandemic pharmacy will need to have the skills to provide accurate and reliable information on issues, including broad topics such as prevention and lifestyle to fight “syndemic” (two or more factors that work together to make a disease worse) and “infodemic” (too much information including false or misleading information during a disease outbreak).

## 1. Introduction

Given their widespread distribution over the territory and extended opening hours, community pharmacies are among the most easily accessible health services serving the public, in particular, for minor problems or general health advice [1,2].

Community pharmacies are, therefore, strategically important structures with the potential to offer a range of public health services. This aspect, already acknowledged in 1994 by the World Health Organisation which described health promotion as a key activity of the community pharmacist [3], is particularly important as community pharmacists come into contact with those sectors of society who need these services most and can have a major impact on disparities in healthcare [4]. Therefore, community pharmacies are being encouraged to expand their range of services from the simple supply of medication to the provision of patient-centred services [5]. Given the challenges of healthcare disparity, this change of role is potentially significant and will be even more so in the future as a consequence of the COVID-19 pandemic (COronaVIrus Disease-19). The fact is that this emergency is now regarded not only as an epidemiological phenomenon but as a “syndemic” or synergistic epidemic.

The term “syndemic” generally refers to an exacerbation of health, environmental, social, or economic problems ensuing from a synergic interaction of two or more transmissible or non-transmissible diseases. This is characterised by serious repercussions, in particular, among those sections of the population that may be regarded as underprivileged. The term was first coined by Merill Singer in the mid-1990s [6]. The word is a combination of the ancient Greek word “synergos”, meaning the union of two or more factors leading to an exacerbation of their impact, and “demos”, which means people [7]. 

In November 2020, Richard Horton, chief editor of The Lancet, pointed out that while the measures taken by world governments centred on checking the spread of COVID-19 by blocking its transmission pathways, the growth in the number of cases demonstrated that, in certain contexts, two different categories of disease interacted: infection with the serious respiratory syndrome known as COVID-19 and a series of non-transmissible diseases such as diabetes, cancer, cardiovascular disease, and chronic respiratory diseases. Therefore, this is not only a pandemic, but a syndemic in that the virulence of the virus increases the prevalence of cases of non-transmissible diseases in the area in which it spreads [8]. 

This is where community pharmacies come into their own: not only safeguarding public health but, moreover, taking over the care of patients and participating actively in reducing health disparity [9,10].

Since the first reported case of the new coronavirus in Wuhan, Hubei province in China, in December 2019, the role of the community pharmacy has become ever more important [11]. Italy was one of the first countries to be struck by this new disease and, indeed, the Italian Cabinet of Ministers had already declared a state of emergency on 31 January 2020 [12], long before the World Health Organisation classified COVID-19 as a global pandemic on 11 March 2020. The country, in general, and specifically healthcare workers, faced a challenge that few had ever been called to deal with before [13].

Amongst the healthcare workers most deeply involved in fighting this epidemic were pharmacists. Almost immediately, but especially from February 2020, access to doctors’ surgeries was highly restricted, with an official press release from the Italian Federation of general practitioners and the Ministry of Health, patients were invited to consult their doctors by telephone and not to go in person to doctors’ offices or health services providing first aid [14]. Consequently, the only healthcare professionals freely accessible to the public were pharmacists. This led to a significant increase in the number of people entering community pharmacies to purchase products such as personal protection equipment, but also to receive clarification and information about the new disease [13]. 

Community pharmacists can play a fundamental role in the community’s or the country’s emergency response and the implementation of preventative and public health measures [1]. For this very reason, community pharmacies remained open and operational throughout the lockdown imposed in many parts of the world with the spread of the new virus. The same happened in Italy, and the critical role of the pharmacies in serving their customers is shown by the fact that the average daily number of entries to community pharmacies in Italy remained constant from the onset of the pandemic and throughout the year with an average of approximately 170 entries/day. 

Given the above and the major impact of COVID-19 in social terms, the purpose of this article is to analyse the evolution of the relationship between community pharmacies and their customers during the different phases of the pandemic over the course of 2020 and to understand which strategies should be implemented in future with a view to curbing the syndemic repercussions of this phenomenon.

## 2. Materials and Methods

The data collection period was from May 2020 to December 2020 in Italian community pharmacies recruited on a voluntary basis. Pharmacists administered a questionnaire, developed in collaboration with a team led by a psychologist, to all customers that agreed to participate. The interviewers were trained by experts to administer the questionnaires as they were built to avoid any influence in the responses of the interviewees: hence, this factor could be ruled out in the survey. The same questionnaire was also available online on social network sites such as Facebook and Instagram and could be completed by anyone interested in taking part.

The questionnaire (Table 1) was in four sections.

Section 1 concerned the pharmacy administrating the survey and classified the pharmacy as either urban or rural (in Italy, pharmacies in centres with a population of fewer than 5000 inhabitants are rural, while those in larger population centres are urban [15]). Respondents also provided some personal details such as age and gender. 

Section 2, made up of 7 questions, was designed to obtain information about the customer’s perception of pharmacies during the pandemic. 

Section 3 dealt with the prospects for the role of community pharmacies. 

In Section 4, the respondent could provide any other comments or suggestions regarding their relationship with the pharmacy during the pandemic.

Questions 4, 7 and 8 were multiple-response questions. The same questions in Sections 1 and 4 also had an open-ended response option. Questions 1, 5 and 9 were questions based on a satisfaction scale. Questions 2, 3 and 6 were polar questions. 

The data were categorised by three time periods for the purposes of analysis. The first period (Period 1) was the months of May 2020 and June 2020; the second period (Period 2) ran from July 2020 to September 2020 inclusive, while the final period (Period 3) was from October 2020 to December 2020 inclusive. These three periods in Italy corresponded to the first wave of the SARS-CoV-2 epidemic, the successive decline in the number of detected cases, and, finally, the second wave of infections respectively. 

Under current legislation in Italy, approval by an ethics committee was not required because the questionnaire was anonymous, personal data were not collected, and there was no way to trace the answers back to a specific respondent.

Descriptive statistics were performed. The comparison between the proportions was performed by Pearson’s chi-square test or Fisher’s exact test. A *p*-value less than 0.05 was considered statistically significant. Statistical analysis was carried out using STATA^®^14 (StataCorp. 2015. Stata Statistical Software: Release 14. College Station, TX, USA: StataCorp LP).

## 3. Results

The results are reported according to different sections of the questionnaire for readability.

### 3.1. Section 1

Participating pharmacies totalled 26, distributed over 8 Italian regions: in the north of Italy (15 in Piedmont and 4 in Veneto), in central Italy (1 in Emilia Romagna and 1 in Lazio) and in the south (1 in Basilicata, 1 in Campania, 1 in Puglia and 2 in Sicily). The total number of respondents was 502, of whom 88 participated in the online survey. The respondents were mainly from the north of Italy, female, and the average age was 49 years, although the sample had subjects of a wide range of ages (interquartile range 31–61 years). With regards to the participants in the online survey only, the average age was 25 years. The majority of the customers (74%) completed the questionnaire in an urban pharmacy, while the majority of the questionnaires were completed in Period 3 of the emergency. The detailed characteristics of the sample are reported in Table 2.

### 3.2. Section 2 

Regarding the role of the pharmacy during the pandemic, 70% of respondents rated this as extremely useful; 58% of customers had entered a pharmacy to obtain information regarding COVID-19 (this percentage rises to 61% in the 31–65 years old age group—differences between age groups were not statistically significant) and in 98% of cases, the requested information was provided. Amongst those who had used a pharmacy as a source of information, the survey revealed a difference between rural and urban pharmacies: 61% of respondents interviewed in an urban pharmacy declared that they had used the pharmacy as a source of information, whereas in rural pharmacies, only 41% of interviewees had done so (*p*-value < 0.05). Moreover, customers who entered the pharmacy to obtain information did so principally in the first two periods of the emergency, i.e., between May 2020 and September 2020. In Period 3, there was an increase in the percentage of customers (45%) who had not used the pharmacy for this purpose (*p*-value < 0.05 calculated by comparing the Period 3 data with the data obtained by combining the data of Period 1 and Period 2). 

Regarding the supplying of PPE, interesting differences were found (*p*-value < 0.05): 73% of respondents felt that the pharmacy had been useful in supplying PPE to the public in Period 1, 85% in Period 2 and 88% in Period 3, so the growth was evident through the three periods.

As for the effects of the restrictions introduced by the emergency regulations in Italy during the pandemic, what respondents found most disruptive was the limits to freedom to move around in the pharmacy and the lack of face-to-face contact with the pharmacist. Considering the online survey only, the most strongly felt disruption was the cancellation of services normally available in the community pharmacy. Comparing the answers to this question across the three age groups, significant differences were found (*p*-value < 0.05). Those who complained about the loss of services were the younger age groups i.e., aged between 18 and 30 years; this was the same for online and in-pharmacy respondents. Interviewees over 65 years complained about not being allowed to move about freely in the pharmacy. Comparing, instead, the different time periods of the survey, a *p*-value of >0.05 was found. The greatest disruption encountered by respondents in Period 1 was the impossibility of moving freely about in the pharmacy; the suspension of services in Period 2 and the lack of face-to-face contact with pharmacists in Period 3.

The new services provided by pharmacies such as home delivery, the option of not going to the family doctor to collect the prescription and phone consultation were considered useful by the majority of respondents and no significant differences were found between rural or urban settings (*p*-value > 0.05): in both locations, 68% of customers regarded access to these services as extremely useful. This figure fell to 60% among the online respondents (*p*-value question 5 in-pharmacy vs. online < 0.05). Considering the differences across the three different periods (*p*-value < 0.05), the new services were considered most useful in Period 3 of data collection, when 70% of customers defined them as extremely useful, compared with Period 1 (61%) or Period 2 (62%). 

Overall, 87% of customers declared that they felt safe entering a pharmacy, but some differences can be noticed across the three periods (*p*-value < 0.05): the percentage was 92% in Period 1; 75% in Period 2 and 88% in Period 3.

As far as criticalities are concerned, 19% of respondents complained about delays in supplying products; 18% were critical of the poor coordination between their family doctor and pharmacist, while 17% felt that prices were too high. Breaking down the data by period, a *p*-value > 0.05 was found, and these figures were at 26%, 21% and 23% respectively in Period 1, but fell to 17%, 18% and 15% respectively in Period 3. Examining this question specifically for urban pharmacies, both delays in supplying the products, and poor coordination between family doctors and pharmacists were criticised by 16% of customers. These figures rose to 32% and 28% in the more specific case of the 18 to 30-year-old age group.

### 3.3. Section 3 

Regarding the future and how the pharmacy could build on the experience of the emergency, the most common options selected by interviewees were the provision of services (25%) and improved collaboration between family doctors and pharmacists (23%). These options were the most popular in percentage terms regardless of age, period or whether the questionnaire was completed in a pharmacy or online. In addition, the home delivery option was also frequently selected (23% of respondents online, against 9% and 10% respectively of customers in urban and rural pharmacies). Interestingly, it should be noted that substantially no respondent selected the home-delivery option in Period 1 or Period 2 of data collection, whereas it was selected by 18% of customers in Period 3. In addition, home delivery was most frequently selected (21%) by customers in the 18 to 30 year age group.

**Figure 1:**
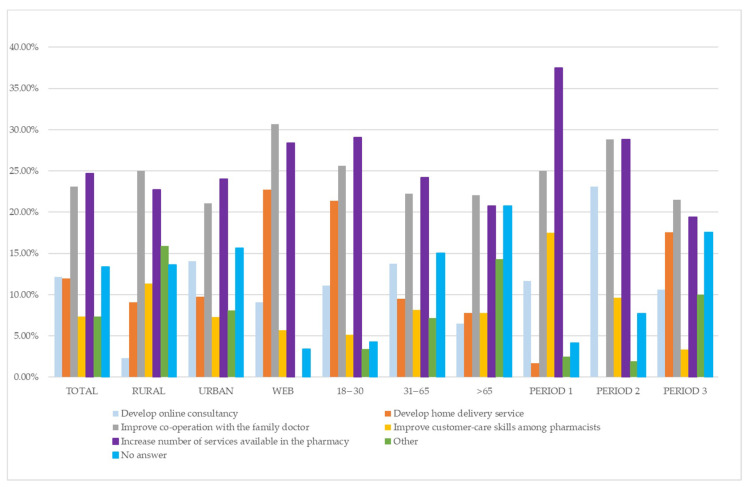
Services that have to be implemented in the future according to the opinion of the interviewed customers.

These results are graphically shown in Figure 1.

**Figure 2:**
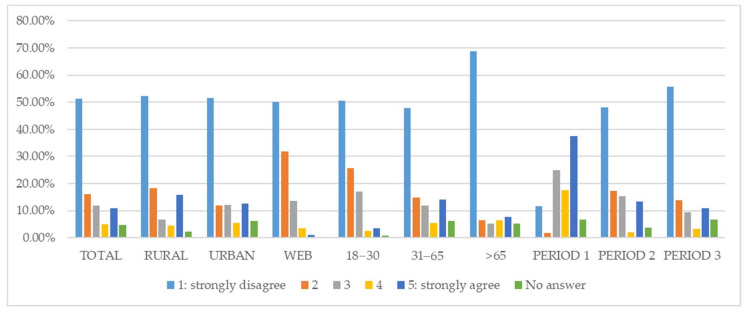
Customer opinion about the prospect that the pharmacy may become an online service in the future.

When questioned about the prospect that the pharmacy may become an online service in the future (Figure 2), at least 50% of respondents strongly disagreed with the proposition, regardless of the source of the questionnaire. This percentage rose to 56% among respondents in Period 3 and reached 69% among the over-65 year age group. Differences observed within the source of the questionnaire, period and age groups were statistically significant (*p*-value < 0.05).

### 3.4. Section 4 

Thirty-nine customers completed Section 4 of the questionnaire. The answers allowed us to detect that community pharmacists’ activity has been considered excellent. Comments showed positive feedback except for the case of a customer reporting that the approach of a pharmacist was not thought to be courteous. All other comments underlined the fact that the community pharmacy was considered as a reference point during the pandemic. Indeed, the pharmacy role was described as “fundamental”, “excellent”, the pharmacist was a “hero”, “point of reference” and the community pharmacy was “an irreplaceable connection between NHS (National Health Service) and citizens that should be strengthened even more as a presence in the territory, to spread good and correct health information among those people who could not access it in any other way”.

All data relating the customers’ answers to Sections 2 and 3 are available in the supplementary material “questionnaire data table”. The answers provided in Section 4 are reported in the supplementary material “answers to Section 4 question”.

## 4. Discussion

The results obtained from the survey confirm a generally high level of satisfaction with pharmacies among customers and a substantial appreciation for the role of community pharmacies.

Some variables such as the biographical factor (in particular age), the environmental factor (differences between rural and urban pharmacies) and the time factor (with respect to the three research periods) have been assessed to better clarify the importance that customers attributed to the relationship between the customer and community pharmacists in the emergency phase.

### 4.1. The “Age Factor”

Traditionally, in pre-pandemic times, the typical customers of pharmacies could be divided into two categories: the over-35s, who purchased non-medicinal products, and the over-60s, who purchased medicines [16]. However, the survey shows a cross-sectional interest in the world of pharmacies generated by the pandemic with interesting variations based on age. The customer profile that emerges for young respondents in the 18–30 years age group, including those that responded online, highlights particular attention to the theme of services as an important added value in this emergency, and even more so for the future. On the other hand, these respondents were particularly critical of delays in being served owing to limits on entry to pharmacies during the lockdown because of the restrictions in force. Similar levels of attention to services are evident in the age groups that traditionally use pharmacies i.e., the 31–65 years age group. This age group merits closer study in terms of future developments as this age group has discovered, and in many cases has become accustomed to online shopping: this has highlighted the difference in prices: higher in pharmacies, which is considered a critical factor and may discourage purchases in pharmacies. The over-65 years age group, who are more accustomed to using a pharmacy, were more critical of factors such as the lack of freedom of movement in the pharmacy and, equally, poor co-ordination between the pharmacist and family doctors. These are areas for improvement in the future; while the digital prescription, introduced as an emergency measure during the pandemic, improved the situation, it is clear that a major reform of the territorial health system is necessary to cater to the more senior age groups.

### 4.2. Rural vs. Urban Pharmacies

On the other hand, rural pharmacies, compared with urban pharmacies, differed substantially in terms of their use as a source of information. In particular, in the initial phase of the epidemic (Period 1), fewer enquiries were addressed to rural community pharmacists than those who work in an urban setting. It is likely that the reason for this is that the emotional impact of the pandemic was much lower in rural areas than in urban centres. However, in both areas, the respondents agreed, especially in Period 1, on the prevalence of delays in supplying personal protection equipment given the shortage of this material on the global market.

### 4.3. Comparisons between Periods

However, the most interesting finding to come out of the survey was the change in perception among the customers over the three periods, that is, between May and June 2020 (Period 1), immediately after the first lockdown, the summer period between July and August 2020 (Period 2), in which there was an easing of the restrictions, and the so-called second wave between September and December 2020 (Period 3). Indeed, for the entire duration of the period analysed, there was no evidence of significant differences in areas such as requests for information at pharmacies. However, from a sociological viewpoint, the data from the three time periods revealed a significant emotional evolution in customers. In particular, in Period 1, the population was driven by strong primary needs (for example, the sourcing of products and protection equipment) and their survival instinct was mixed with a sense of national identity and the need to keep hopes high (symbolised by banners extolling hope for the future or the national anthem being sung from balconies). The easing of emergency measures in the summer season (Period 2) brought about a shift in attention amongst interviewees. The urgent primary needs associated with the initial phase of the pandemic gave way to more purely relational and professional qualities as being the distinctive features of a pharmacy. In the second wave (Period 3), the realisation of the persistence of the pandemic led to an increase in fear for the future which shifted attention to the search for safety and the need for advice on preserving health. In this context, even more markedly, the theme of co-ordination between pharmacist and family doctor, as well as services, including the use of online solutions, stood out as the impelling necessity for patients who were highly intolerant, based on personal experience, to the hypothesis of complete digitalisation of the patient-pharmacist relationship.

### 4.4. The Importance of the Open-Ended Questions

Open-ended questions represent the most effective means of activating a brainstorming process in the interviewed subject. This allows the experimenter simultaneously to sense and comprehend the cognitive and emotional factors expressed in an opinion or viewpoint [17]. To achieve the objectives of this research, it was considered opportune to complete the investigation by proposing a series of stimulus situations that encouraged the interviewees to express their sentiments (a general feeling, attitude, or opinion about something [18]), which were denoted as the result of experiences in the pandemic and experiences of their relationship with pharmacies. 

On this subject, two analytical macro-categories could be distinguished: the first, refers to the open-response option “other” contained in questions 4, 7, and 8; the other, with a broader scope, regards Section 4, in which the respondent was asked to provide personal comments or suggestions with regards to the object of the research.

In the first macro-category (“other”), the respondent could enter personal comments on the effects of the restrictions. The answers showed that the quality of customer care offered by pharmacists largely offset the absence of face-to-face contact caused by the social distancing regulations. Such was the customers’ appreciation of this effort that this factor did not feature among the areas in need of substantial improvement in the coming years. Analysing the answers in more detail, it is, however, worth noting the respondents’ negative feedback regarding the access limits, with the resulting queues outside the pharmacy, as well as the difficulties in purchasing PPE. This difficulty, which was very evident during the first wave of the pandemic, gradually improved over time with the greater availability of this material starting from the summer of 2020.

Of greater interest, on the other hand, is what emerged from the final question (Section 4), where the opportunity to express one’s comments or suggestions allowed the respondent to express personal emotional experience. In this context, two levels of analysis were important: feeling and opinion. At the first level, the survey dealt with a pharmacy, and, hence, a pharmacist: this is viewed with greater recognition and appreciation for expressing humanity under difficult circumstances: terms such as empathy and helpfulness emerged in describing the behaviour of this figure during the pandemic, especially in light of the sense of disorientation exacerbated by the difficulty in gaining access to healthcare professionals and health centres, unlike community pharmacies [9,10]. Turning to the term opinion: on one hand, the research confirmed the perception that the pharmacy was an irreplaceable institution whose presence represented then, and now, as a reference point for members of the public who experienced difficulties in accessing healthcare structures; on the other hand, respondents expressed a desire for greater cooperation between pharmacists and family doctors, which is strictly limited by current legislation in Italy [19]. This must change in order to define a new perimeter within which to develop an integrated service between the various sectors of the NHS.

From the obtained results, a profile of the customer emerged who, regarding all the concerns generated by the pandemic, found primarily in the community pharmacist a human comfort.

### 4.5. The Future? Between Infodemic and Syndemic

It is indisputable that the COVID-19 pandemic has had the impact of a tsunami in the health sector, but also at an economic and social level, its effects are destined to persist for many years to come [11]. The emergency nature of this pandemic is consequently forcing the health services responsible for combatting it to rethink healthcare governance [20].

In this context, the community pharmacy, which has always been a source of accurate and science-based information, serving as a link between the “vertical” healthcare system (i.e., the hospital, which is there to deal with dangerous or serious pathologies) and the “horizontal” healthcare system tied to popular knowledge, has demonstrated its ability to react to the needs of the population. This was evident even in the most challenging initial phases of the pandemic when a sense of anxiety reigned, generated by its unknown nature [21,22,23].

In the near future, the demand will intensify for community pharmacies to assume responsibility for patients while still providing adequate healthcare, with a strategic focus on promoting health and preventing pathologies. This strategy will go some way towards reducing disparities within the population [4,9]. It will also be fundamental to pushing back against not only the syndemic phenomenon but another very relevant concern: infodemia.

Infodemia is defined as an excessive quantity of unverified information in circulation during a disease outbreak which makes it difficult to form a correctly informed opinion on certain topics due to the difficulty in finding reliable sources of information. With this definition of the problem, the World Health Organisation wished to underline the risks for a global society in an era of social networks and the deformation of reality through the comments in the online community based on real, or, all too often, false or misleading facts. In this case, “do it yourself” healthcare may take on connotations that are quite perilous if they turn into “home-made solutions to the health emergency” with all the imaginable consequences [24].

Both infodemias and syndemias, which have become reality as a consequence of the current pandemic [25], in light of the evidence that has emerged in the present research, will be particularly important in the near future, especially if one considers that their sum could exacerbate disparities. 

The post-pandemic pharmacy will increasingly have to strengthen its ability to provide accurate and reliable information on issues, including broad topics such as prevention and lifestyle in general to fight the infodemia-related concerns [21].

### 4.6. Limitations

The limitations of this study may have affected the results. 

Firstly, the fact that pharmacies participated in the survey on a voluntary basis may reflect that participating pharmacies were better than others in terms of services, personnel, and facilities and, therefore, the results about the quality of services in the pharmacy may have been overestimated.

The number of pharmacies involved (26 out of the 19,000 in Italy) may not be considered representative of the whole population of Italy.

Another limitation is the fact that customers entering a pharmacy are intrinsically more confident in pharmacies than the overall population.

Another limit that should be considered concerns the construction of Question 6 which may have influenced the answer.

## 5. Conclusions

The COVID-19 pandemic represented a crisis to which the NHS had to adapt rapidly and deliver an immediate response. During the last decades, the role of the community pharmacist has substantially changed: the pharmacy has shifted from being products-based to being patient-centered. As a result of the pandemic, it is probable that a new era in the history of pharmacies has begun, with community pharmacists acquiring more specific professional standing.

What emerges, indeed, is the priority to monitor the situation on the ground at a grassroots level and control any changes: a task that can certainly be entrusted to the branch of the healthcare service ideally suited to this end: local medicine, of which the community pharmacy is an essential element.

The repeated reference of the respondents to the theme of services, as well as the need for greater co-ordination between the pharmacist and the family doctor, is of particular importance in the future development of the community pharmacy, which must be capable of responding to new health requirements focusing on prevention and lifestyle. 

In the near future, the community pharmacy will be called upon to face and overcome a new challenge generated by the pandemic, and which was completely unforeseeable only a year ago: the struggle against infodemia and syndemia.

Only if the community pharmacy will be able to respond to the new challenges will it truly be effective in the protection of citizens, especially those who are disadvantaged.

## Figures and Tables

**Table 1:** Questionnaire.

**Section 1**	
Pharmacy: ◦ Urban ◦ Rural	City: _______________________________ Date of completion: ____________
Respondent age: ______________	Respondent gender: ◦ Male ◦ Female
**Section 2**	
In this phase of the COVID-19 emergency, how useful do you rate the role of pharmacies overall? 1: not at all useful 5: extremely useful	◦ 1 ◦ 2 ◦ 3 ◦ 4 ◦ 5
2. In this phase of the COVID-19 emergency, have you used a pharmacy to obtain information about COVID-19? (Symptoms, safety precautions, use of PPE ^1^, etc.)	◦ Yes ◦ No
If so, did you receive the information requested?	◦ Yes ◦ No
3. Do you feel that the pharmacy was useful in supplying the public with essential PPE such as masks, gloves, etc.?	◦ Yes ◦ No
4. Given the safety measures in place, what did you find to be the biggest disruption of services in the pharmacy?	◦ Loss of face-to-face contact with the pharmacist ◦ Restricted access to services (blood pressure, Holter, point-of-care test, etc.) ◦ Restricted freedom of movement in the pharmacy ◦ Loss of access to special health days ◦ Other____________________
5. How useful do you rate the new services provided by the pharmacy in this emergency phase (home delivery, possibility to not go to the family doctor to collect the prescription, phone consultation)? 1: not at all useful 5: extremely useful	◦ 1 ◦ 2 ◦ 3 ◦ 4 ◦ 5
6. Do you feel that a pharmacy is a safe place or that it may be a site of infection like the emergency department of a hospital?	◦ Yes, I feel safe ◦ No
7. What was the most negative experience you have had in the pharmacy in this emergency phase?	◦ Unfriendly or distracted staff ◦ Poor co-ordination between my family doctor and the pharmacist ◦ Delays in supplying products ◦ Products are too expensive ◦ Other ____________________
**Section 3**	
8. In your opinion, how can the pharmacy improve its service(s) in the future given your experiences during this emergency?	◦ Develop online consultancy ◦ Develop home delivery service ◦ Improve co-operation with the family doctor ◦ Improve customer-care skills among pharmacists ◦ Increase number of services available in the pharmacy ◦ Other ____________________
9. What do you think of the idea of a completely online pharmacy in the future? 1: strongly disagree 5: strongly agree	◦ 1 ◦ 2 ◦ 3 ◦ 4 ◦ 5
**Section 4**	
Please provide any additional comments or suggestions you have regarding the relationship between yourself and the pharmacy during the COVID-19 emergency	

^1^ PPE: Personal Protection Equipment.

**Table 2:** Number of customers participating in data collection divided by region, period, age, gender, type of pharmacy.

**Region Where the Questionnaire Was Completed**	**Total** **Customers**	**Customers** **Period 1**	**Customers** **Period 2**	**Customers** **Period 3**	**Average** **Age**	**Males**	**Females**	**Gender Not** **Declared**
Basilicata	25	0	21	4	52	12	12	1
Campania	29	0	0	29	44	12	16	1
Emilia Romagna	22	0	0	22	57	5	17	0
Lazio	30	0	30	0	54	7	18	5
Piedmont	155	120	0	35	52	64	91	0
Puglia	34	0	1	33	53	21	13	0
Sicily	38	0	0	38	49	13	25	0
Veneto	81	0	0	81	57	15	62	4
Online	88	0	0	88	25	17	71	0
TOTAL	502	120	52	330	49	166	325	11
**Type of Pharmacy in Which the Questionnaire Was Completed**	**Total** **Customers**	**Customers** **Period 1**	**Customers** **Period 2**	**Customers** **Period 3**	**Average** **Age**	**Males**	**Females**	**Gender Not** **Declared**
Rural	44	22	0	22	56	12	32	0
Urban	370	98	52	220	53	137	222	11
TOTAL	414	120	52	242	53	149	254	11

## Data Availability

All data is contained in the article or Supplementary Material.

## Supplementary Materials

All data relating the customers’ answers to Sections 2 and 3 are available in the “questionnaire data table”. The answers provided in Section 4 are reported in the document “answers to Section 4 question”.

## Submission Declaration

This manuscript is an original paper, has not been published before, and is not under consideration for publication in another journal at the present time.
