# Supplementary material for: The Relationship between Customers and Community Pharmacies during the COVID-19 (SARS-CoV-2) Pandemic: A Survey from Italy
Source: Int J Environ Res Public Health. 2021 Sep 11;18(18):9582. doi: 10.3390/ijerph18189582 (PMC8470071; doi:10.3390/ijerph18189582)
Supplement: Supplementary file 1 [file ijerph-18-09582-s001.zip › Answers to section 4 question_supplementary matherial.pdf]

Answers to section 4 question

Please provide any additional comments or suggestions you have regarding the relationship between the you and the pharmacy during the COVID-19 emergency

1. The human contact of the pharmacists has been fundamental
2. Human contact is essential
3. Empathy and trust in the pharmacist would not be replaceable with any virtual solution
4. Pharmacists have been heroes
5. The relationship has been optimal
6. I have maintained an excellent relationship with the staff and their services
7. The relationship was top
8. Optimal relationship
9. Super availability, seriousness, kindness in all respects
10. Everything was ok
11. The direct contact with the personnel is absolutely important
12. I want to say thanks to my pharmacists
13. Competence, availability, friendliness
14. It has been perfect
15. They have been always present
16. Pharmacy that meets all needs in response to the COVID problem
17. Excellent relationships
18. I am currently very satisfied. I think that in case of further needs they will be able to improve
19. I feel very comfortable and they are all very good
20. It has been an important point of reference
21. Almost always perfect
22. I have not encountered any difficulties and I have always found what I was looking for
23. In this pharmacy I have always found surgical masks
24. Always attentive and helpful staff
25. I did not find significant differences in the pre and post COVID period
26. Essential
27. Pharmacy well available and equipped in this period COVID-19
28. Great professionalism, availability and kindness
29. Everything worked well
30. Excellent relationship
31. My pharmacy has increased availability and services for customers
32. Great availability
33. They are very important
34. I have not had any inconvenience and therefore I am satisfied with the service received
35. Direct contact with the pharmacist is always necessary, especially for consultations
36. They have become more available than hospitals
37. It is true that in this emergency period the tension is stronger on everyone and everyone, but more and more often I find myself dealing with young pharmacists who are increasingly rude. Often even an expression is synonymous with rudeness, especially for those who have to do with the public
38. I went to the pharmacy only in cases of real need and in any case placing more trust in the advice of the pharmacist than in that of the general practitioner
39. The current situation confirms that pharmacy is an irreplaceable connection between Healthcare and citizens that should be strengthened even more as a presence on the territory, to spread good and correct health information among those people who could not access it in any other way. This role of the pharmacy is often taken for granted or even underestimated
